# Supplementary material for: Mitochondria localization induced self-assembly of peptide amphiphiles for cellular dysfunction
Source: Nat Commun. 2017 Jun 21;8:26. doi: 10.1038/s41467-017-00047-z (PMC5479829; doi:10.1038/s41467-017-00047-z)
Supplement: Supplementary file 1 — Supplementary Information [file 41467_2017_47_MOESM1_ESM.pdf]

File Name: Supplementary Information
Description: Supplementary Figures, Supplementary Methods, Supplementary References.

File Name: Peer Review File
Description:

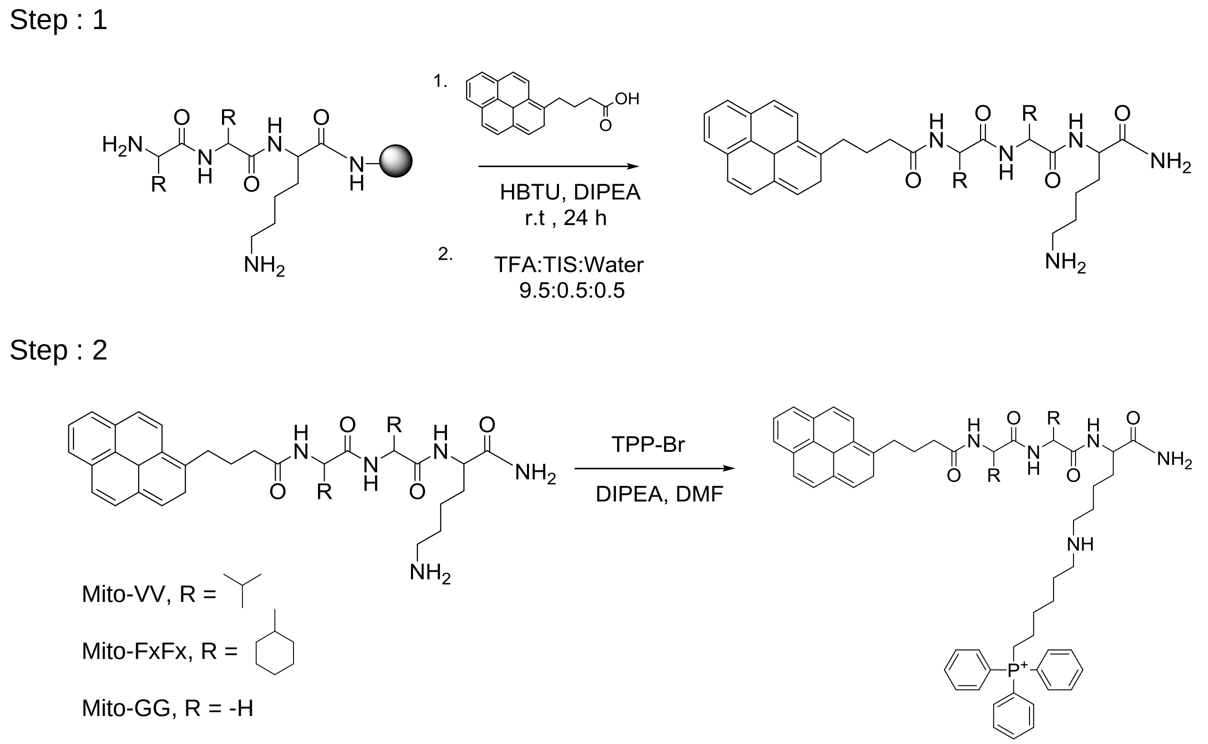

Supplementary Figure 1 | Schematic representation for the synthesis of Mitochondria-accumulating peptides. The peptides have been synthesized by the standard 9-fluorenylmethoxycarbonyl solid-phase peptide synthesis on a 0.25 mmol scale. The synthesized peptides were treated with 1-pyrene carboxylic acid (500 µmol) and *O*-(Benzotriazol-1-yl)-*N,N,N',N'*-tetramethyluronium hexafluorophosphate (HBTU) (500 µmol) in presence of diisopropyl ethyl amine (DIPEA, 500 µmol) and allowed to stir at room temperature for 24 h in DMF. The resin was collected by filtration and washed with dimethylformamide (DMF) to remove unreacted chemicals. The product cleaved from the resin with cleavage cocktail (TFA/Water/Tri isopropyl amine mixture (9.5: 0.5: 0.5)) and products were precipitated in cold ether. Purified by HPLC and confirmed by mass analysis using MALDI-TOF/TOF. To achieve the triphenyl phosphonium (TPP) conjugation, synthesized peptide (0.02 mmol) was treated with 1-hexyl triphenylphosphonium bromide salt (0.04 mmol) with triethyl amine (0.02 mmol) in DMF and allowed to stir for 12 hr at room temperature. The pure product were collected using HPLC, freeze-dried and used for further studies.

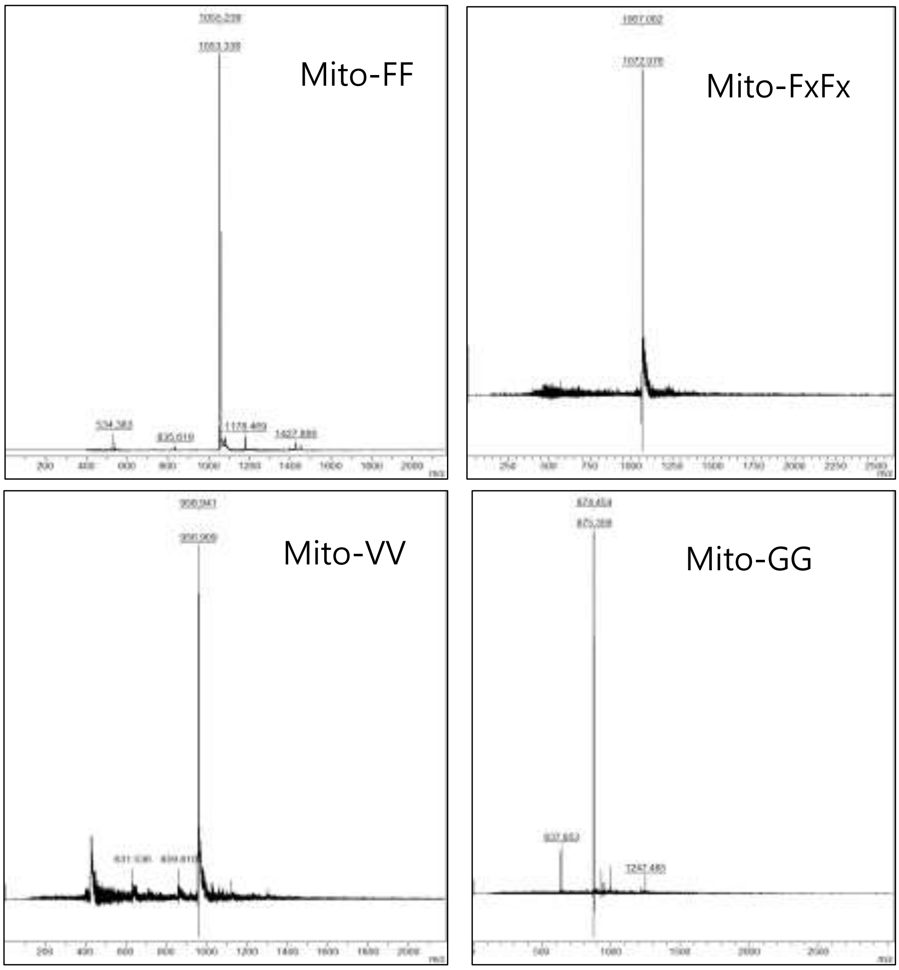

Supplementary Figure 2 | The HPLC purified peptide (ACN/Water mixture, C 18 column) was confirmed using MALDI-TOF analysis. 10 μL of peptide solution in MeOH were mixed with 10 μL of MALDI matrix (α-cyanocinnamic acid) and analyzed after drying. The product was confirmed by the appearance of m/z peak. MALDI-TOF for **Mito-FF**, **Mito-F_xF_x**, **Mito-VV** and **Mito-GG**.

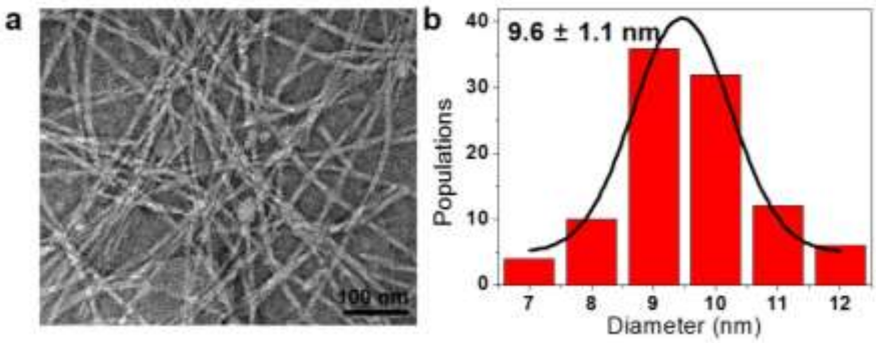

Supplementary Figure 3 | a) TEM image (stained with 2 wt% uranyl acetate) for **Mito-FF** fibers and b) diameter distribution.

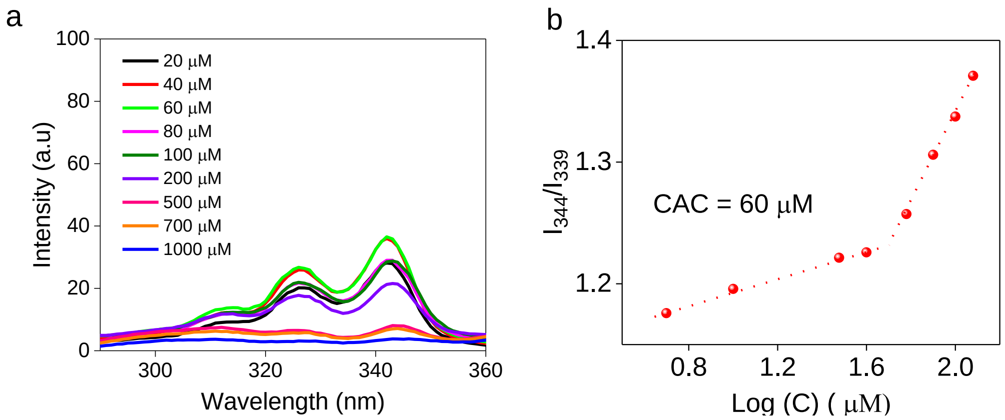

Supplementary Figure 4 | The CAC determination of **Mito-FF** in PBS (10 mM) (a) Steady state fluorescence excitation spectra of **Mito-FF** at 376 nm for pyrene probe on the **Mito-FF** with various concentration in PBS at room temperature. (b) Plot of I_{344}/I_{339} ratio of pyrene excitation in PBS ad a function of Log [**Mito-FF**].

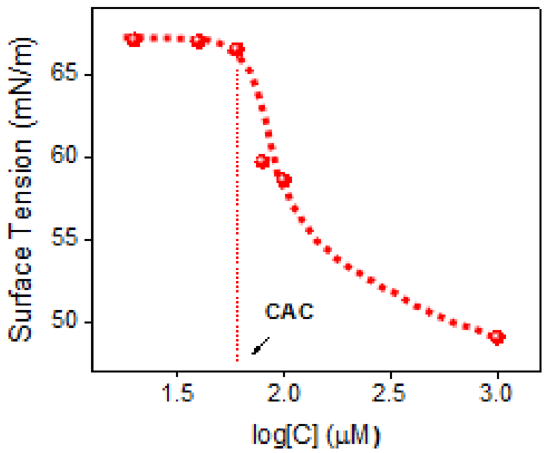

Supplementary Figure 5 | Plot of **Mito-FF** surface tension as a function of Log [**Mito-FF**]. The CAC was indicated by the sudden drop of surface tension value calculated from the contact angle.

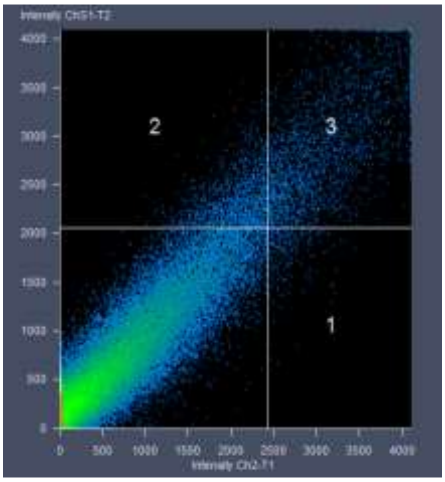

Supplementary Figure 6 | Fluorescent correlation of blue fluorescence of **Mito-FF** and red fluorescence of MitoTracker showing high correlation with Rr of 0.80 which reflect the mitochondrial specificity.

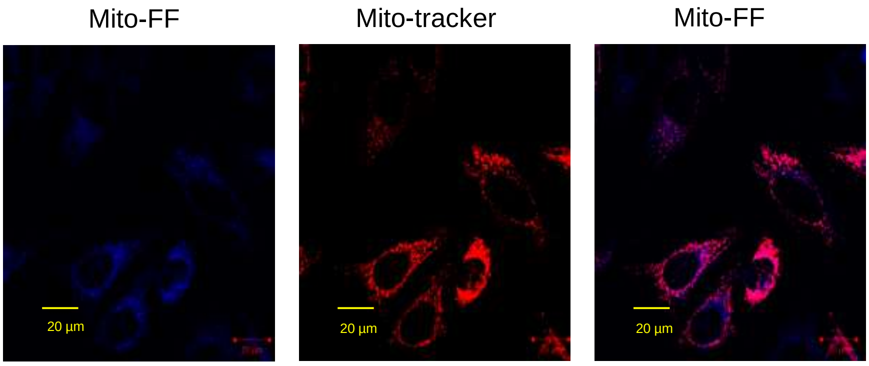

Supplementary Figure 7 | Mitochondrial co-localization of **Mito-FF** and MitoTracker at 4 ^{o}C, indicating that **Mito-FF** enters inside the cell via an energy independent pathway.

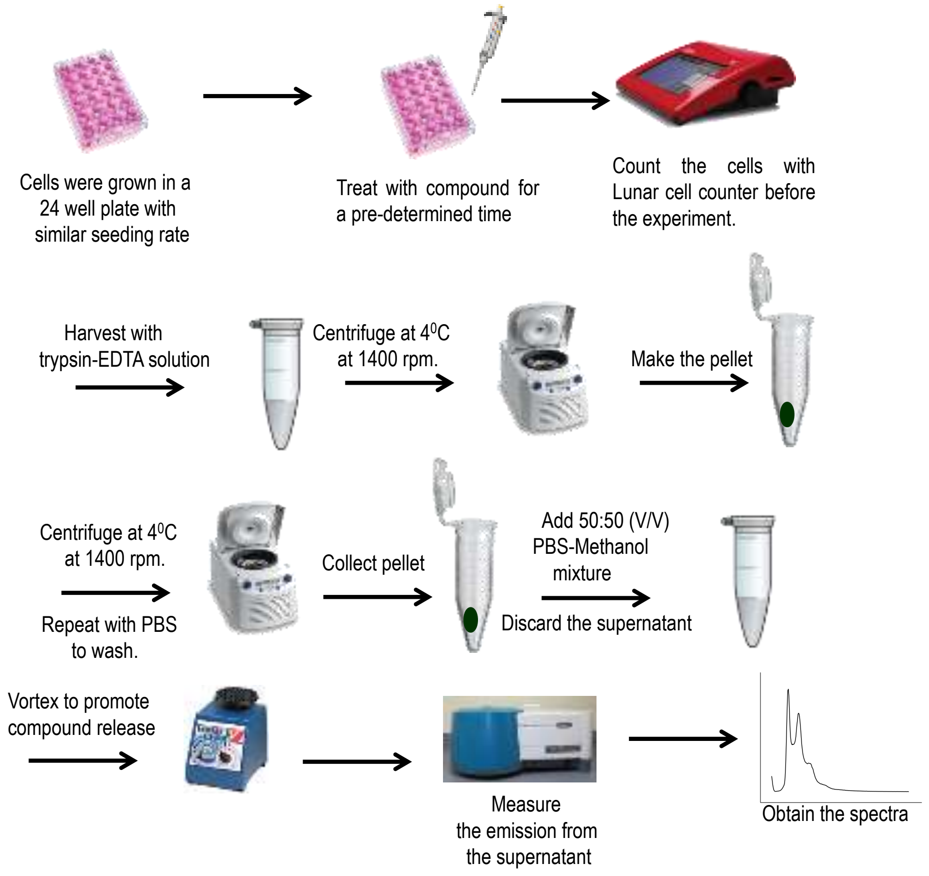

Supplementary Figure 8 | Schematic illustration for the calculation of **Mito-FF** concentration inside mitochondria.

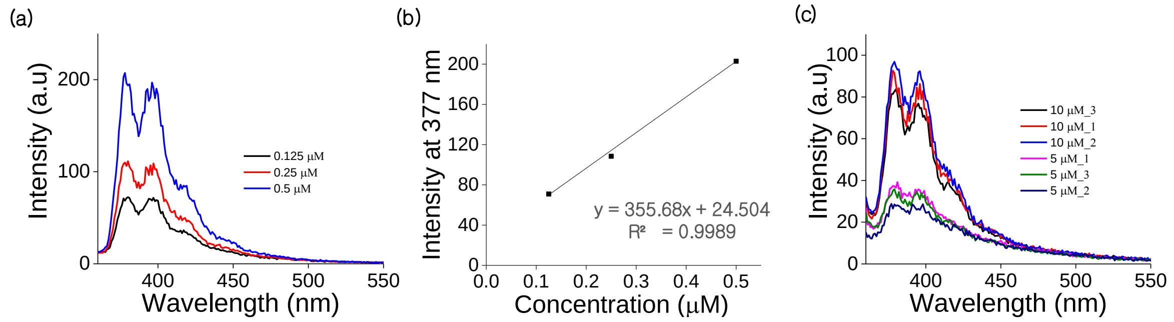

Supplementary Figure 9 | IMC determination for Mito-FF (a) Emission spectra of **Mito-FF** in Buffer/MeOH mixture (1:1) recorded for the generation of calibration plot. (b) Calibration plot of **Mito-FF** from the emission spectra. (c) Emission spectra of HeLa cell lysate (Buffer/MeOH mixture (1:1)) after 3 h treatment with **Mito-FF**.

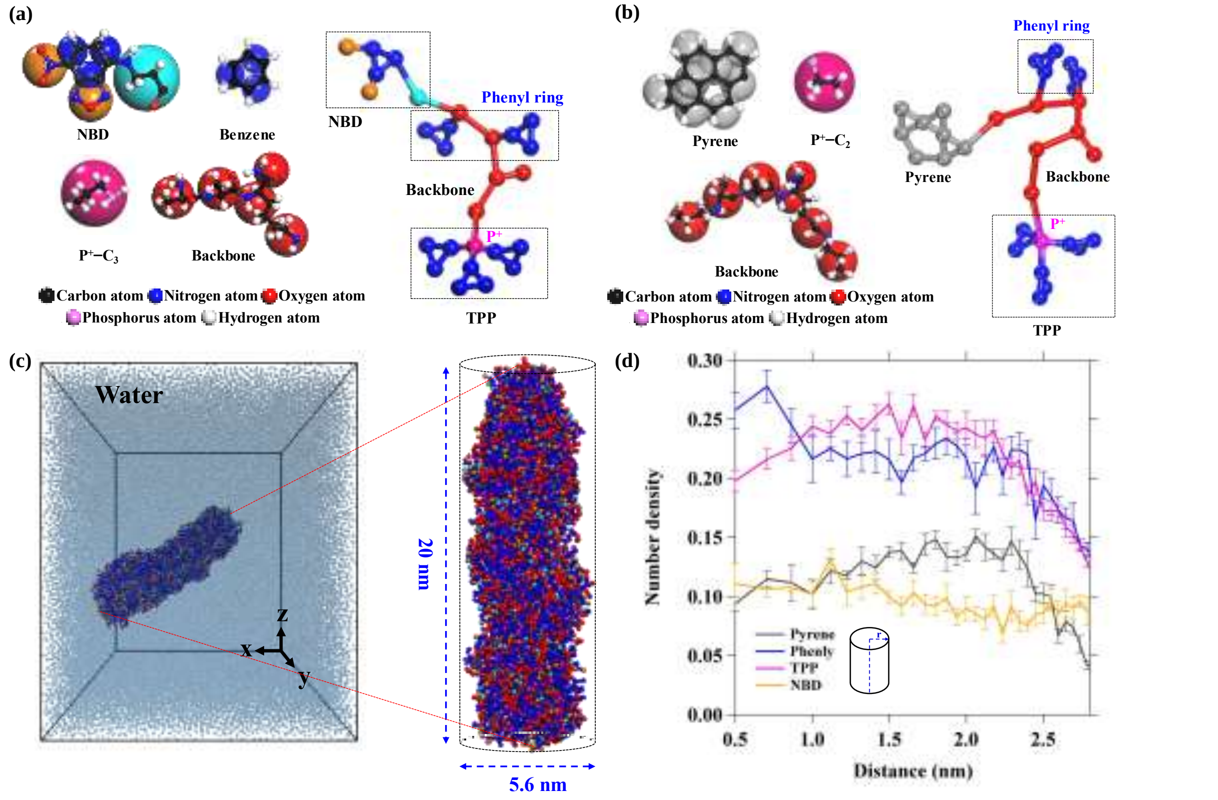

Supplementary Figure 10 | Coarse-grained (CG) model of (a) **Mito-FF-NBD** and **(b) Mito-FF**. Details of simulation method are introduced in the section 1.6. NBD, phenyl ring, TPP and pyrene groups consist of six, six, ten and eight CG beads, respectively. NBD group contains O–N–O, N–O–N, N–C_3–O and benzene ring. Backbone of **Mito-FF-NBD** consists of five CG beads, which are 3 amides and 2 amines, and **Mito-FF** consists of seven CG beads, which are 1 ketone, 3 amides, 2 amines, and 1 alkyl groups. (c) Snapshot of the simulation result of cylindrically self-assembled 174 **Mito-FF-NBDs** and 174 **Mito-FFs** in the box (i.e. 25×25×30 nm^3) filled with water after performing CGMD for 3.6 μs. The cylinder shows effective radius and length of the **Mito-FF-NBD** and **Mito-FF** fibril. (d) Radial number density of four constituent molecules in the fibril from the principal axis of fibril to its surface. The numbers of groups in the same volume of radial shell were counted six times within the final 50 ns of the MD simulation and averaged with a 10 ns interval. Hydrophilic TPP and hydrophobic phenyl groups showed high density compared to others since they were contained in both **Mito-FF-NBD** and **Mito-FF**. High density of phenyl group at the center of fibril indicated the hydrophobic characteristics inside of the fibril. NBD groups were well distributed in that hydrophobic environment within the fibril.

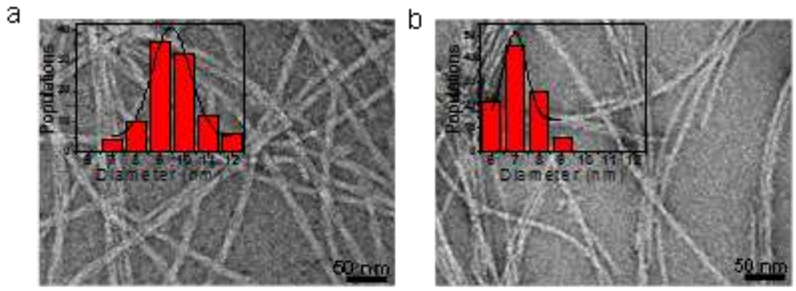

Supplementary Figure 11 | Negatively stained TEM image of a) **Mito-FF** and b) co-assembly of **Mito-FF** (500 µM) and **Mito-FF-NBD** (100 µM) (inset; Diameter distribution graph of nanofibrils. The image was taken with 2 wt% uranyl acetate staining. The TEM images of **Mito-FF** and **Mito-FF-NBD** co-assembly showed a decreased diameter (7.1 ± 0.8 nm averaged over 100 nanofibers) compared to **Mito-FF** nanofibrils alone suggesting that they assemble each other.

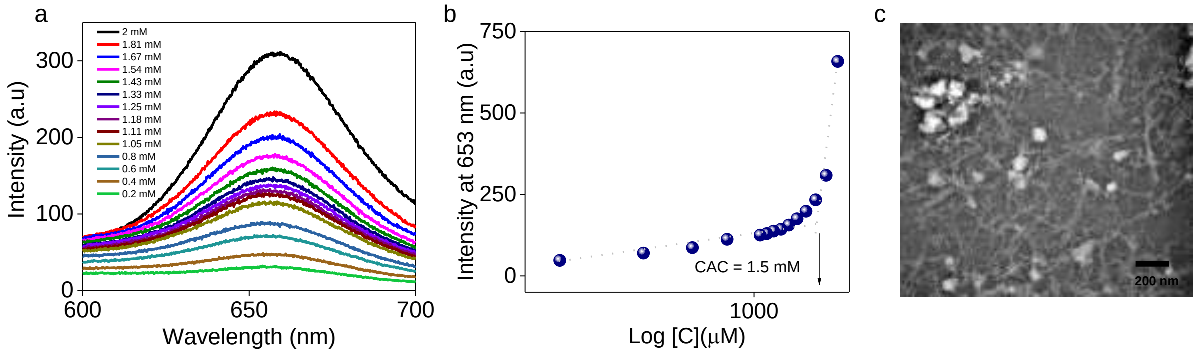

Supplementary Figure 12 | CAC determination for **Mito-FF-NBD** using Nile red encapsulation method. (a) Emission spectra of Nile red at an excitation of 550 nm. (b) Intensity of Nile red emission at 653 nm plotted against log concentration of **Mito-FF-NBD**. (c)TEM images showing the nanofibrils formed by **Mito-FF-NBD** at 2 mM concentration.

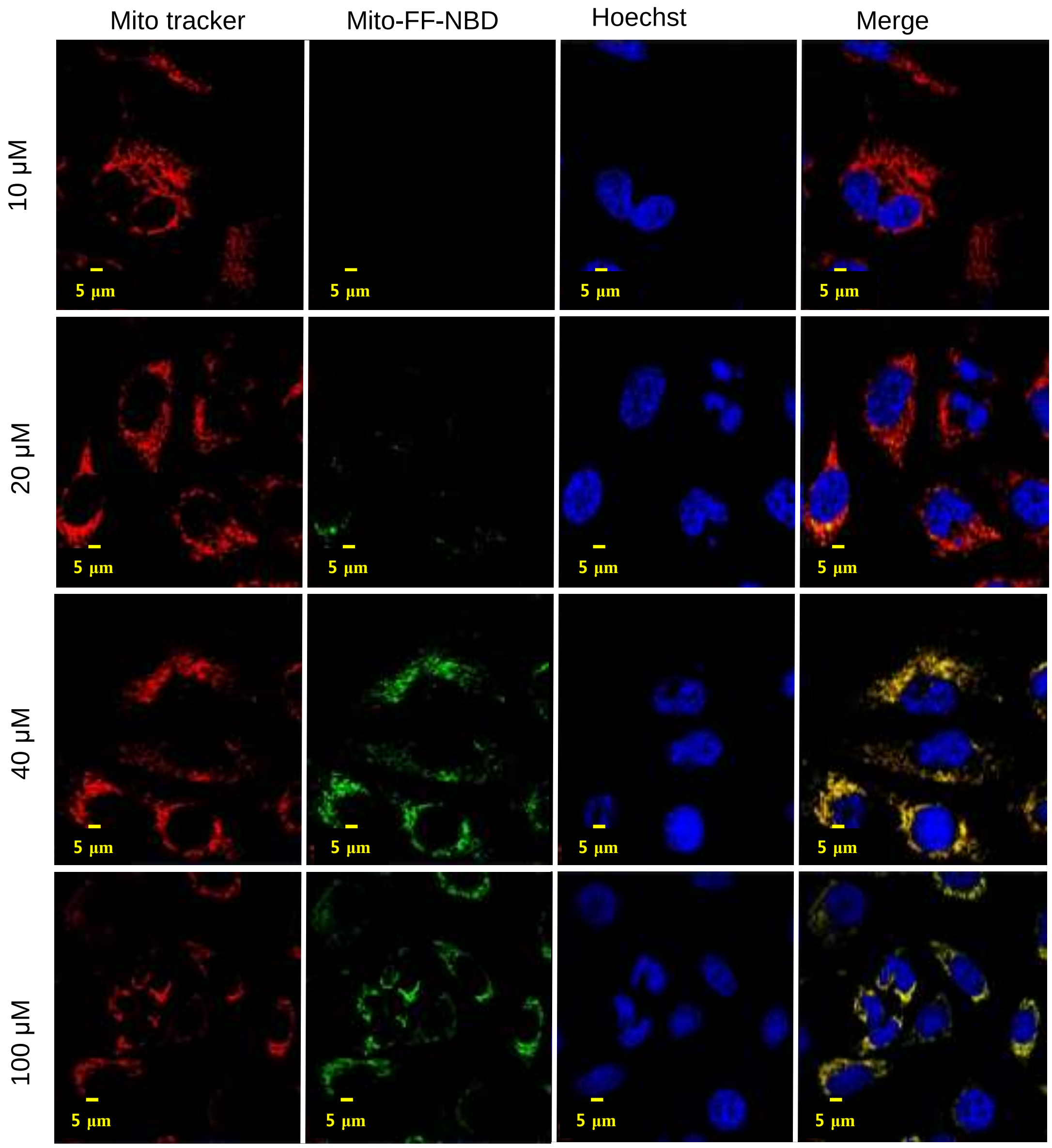

Supplementary Figure 13 | Dosage dependent confocal microscopic analysis for **Mito-FF-NBD.** Blue: Nuclei stain, Red: MitoTracker, Green: Mito-FF-NBD.

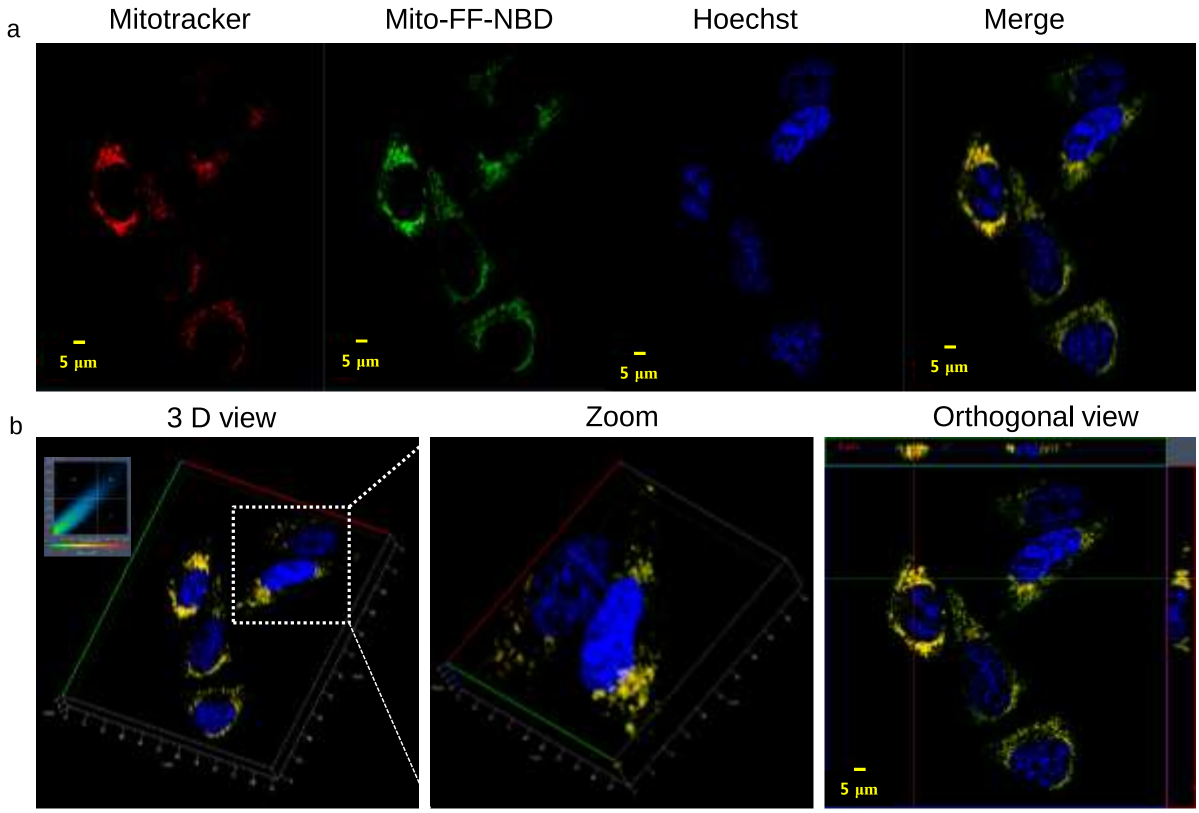

Supplementary Figure 14 | a) 2D image showing co-localization of **Mito-FF-NBD** inside mitochondria and b) 3D localization for **Mito-FF-NBD** (middle: zoom view, right: orthogonal view). Inset: co-localization between mitotracker and Mito-FF-NBD. Blue: Nuclei stain, Red: MitoTracker Red FM, Green: **Mito-FF-NBD** (left)Zoom image (middle) Orthogonal view (right).

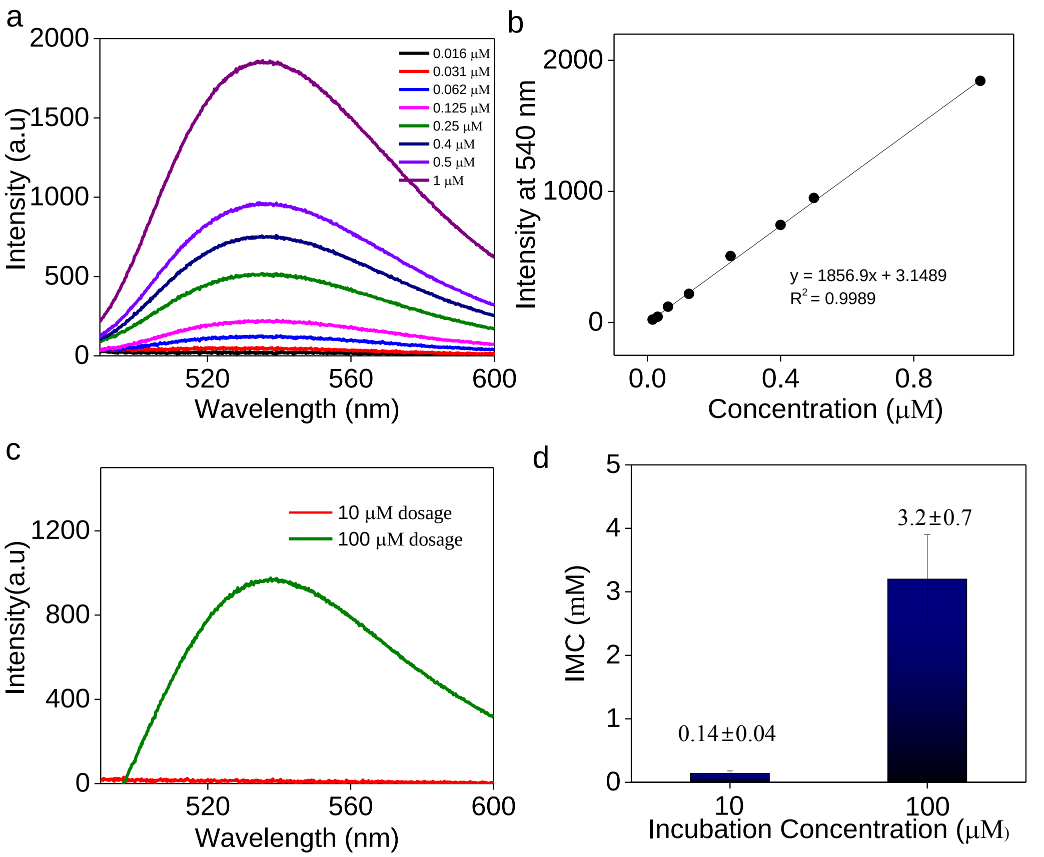

Supplementary Figure 15 | IMC determination for Mito-FF-NBD. (a) Emission spectra of **Mito-FF-NBD** in Buffer/MeOH mixture (1:1) recorded for the generation of calibration plot. (b) Calibration plot of **Mito-FF-NBD** from the emission spectra. (c) Emission spectra of HeLa cell lysate (Buffer/MeOH mixture (1:1)) after 3 h treatment with **Mito-FF-NBD.** (d) IMC of **Mito-FF-NBD** after 3 h treatment with HeLa cells. Data represent mean ± s.d from three independent experiments.

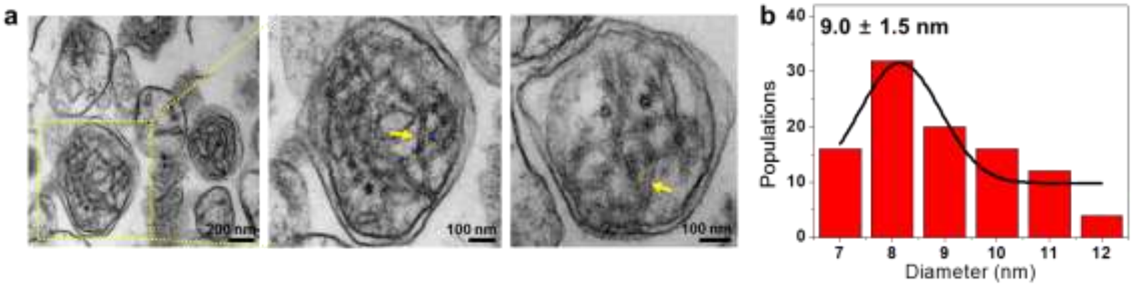

Supplementary Figure 16 | a) **Mito-FF** fibrils inside mitochondria isolated from a C3H female nude mouse brain and b) diameter distribution of **Mito-FF** nanofibrils within the isolated mitochondria

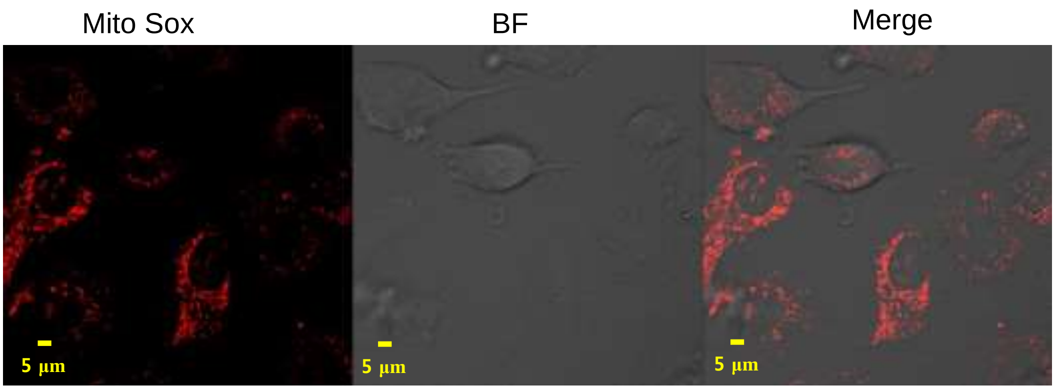

Supplementary Figure 17 | ROS generation inside the mitochondria monitored by the red fluorescence from MitoSOX. **Mito-FF** was incubated for 6 h with HeLa cells and stained with MitoSOX before 10 minute of measurement and analyzed via confocal microscopy. The red fluorescence form MitoSOX confirmed the ROS inside mitochondria.

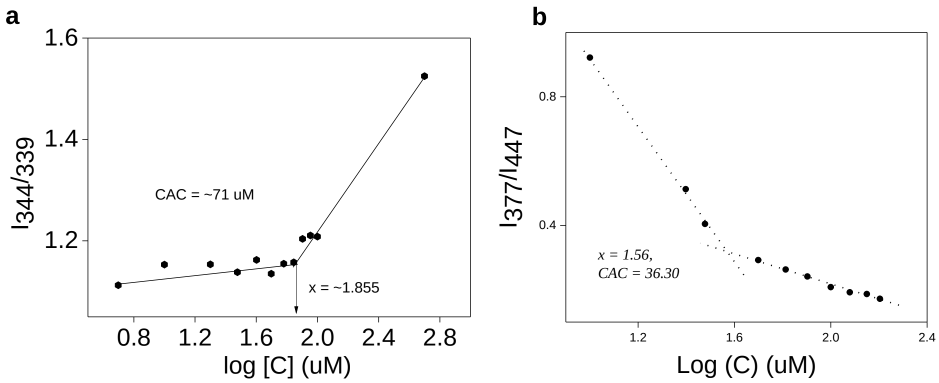

Supplementary Figure 18 | CAC determination of (a) **Mito-VV** (b**) Mito-**$\mathbf{F_xF_x}$ in PBS (10 mM). The CACs for **Mito-VV** were determined by the steady state fluorescence excitation spectra at 376 nm for pyrene probe with various concentrations in PBS at room temperature. Plot of I_{344}/I_{339} ratio of pyrene excitation was obtained in PBS as a function of Log [**Mito-VV**].For CAC for **Mito-**$\mathbf{F_xF_x}$ were determined from the concentration dependent emission spectra of **Mito-**$\mathbf{F_xF_x}$ followed by the obtaining the pyrene I_3/I_1 ratio. The Log [**Mito-** $\mathbf{F_xF_x}$] were plotted against I_3/I_1 (I_{377}/I_{447}).

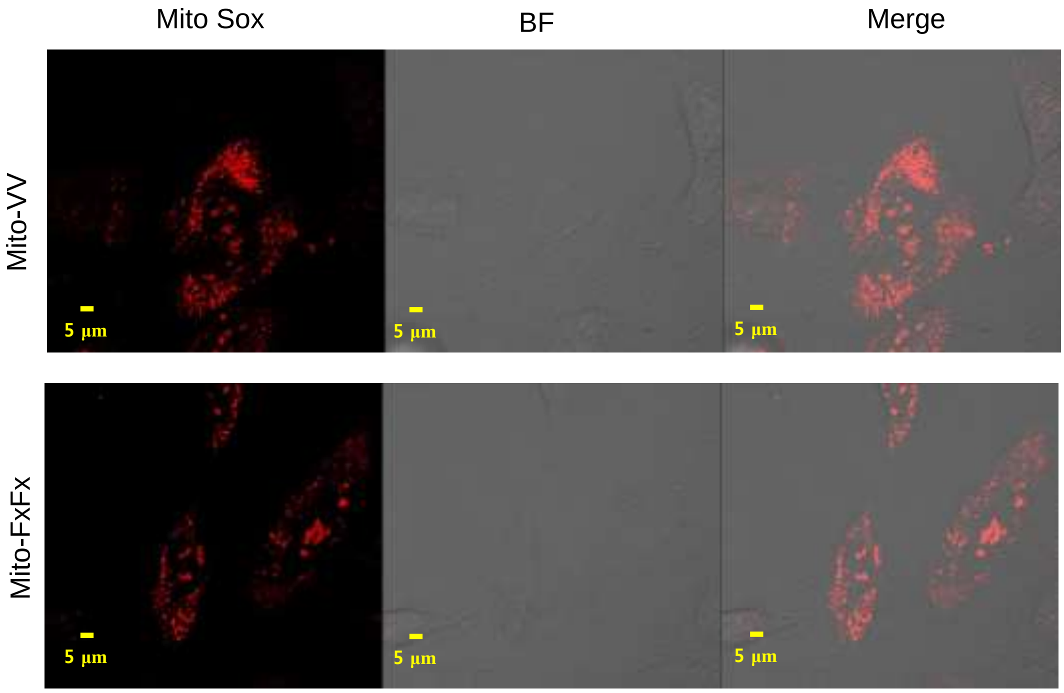

Supplementary Figure 19 | ROS generation inside the mitochondria monitored by the red fluorescence from MitoSOX. **Mito-VV** or **Mito-F_xF_x** was incubated for 6 h with HeLa cells and stained with MitoSOX before 10 minute of measurement and analyzed via confocal microscopy. The red fluorescence form MitoSOX confirmed the ROS inside mitochondria.

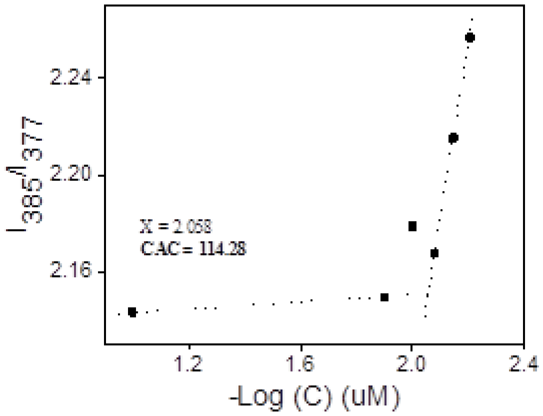

Supplementary Figure 20 | CAC determination of **Mito-GG** in PBS (10 mM). The CAC were determined by the steady state fluorescence excitation spectra of **Mito-GG** at 376 nm for pyrene probe with various concentrations in PBS at room temperature. Plot of I_{344}/I_{339} ratio of pyrene excitation were obtained in PBS as a function of Log [**Mito-GG**].

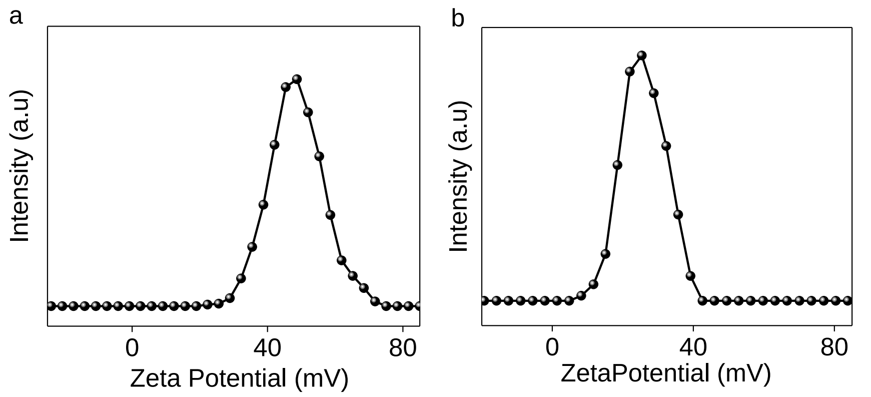

Supplementary Figure 21 | surface charge analysis of Mito peptides. a) **Mito-FF**, b) **Mito-GG.**

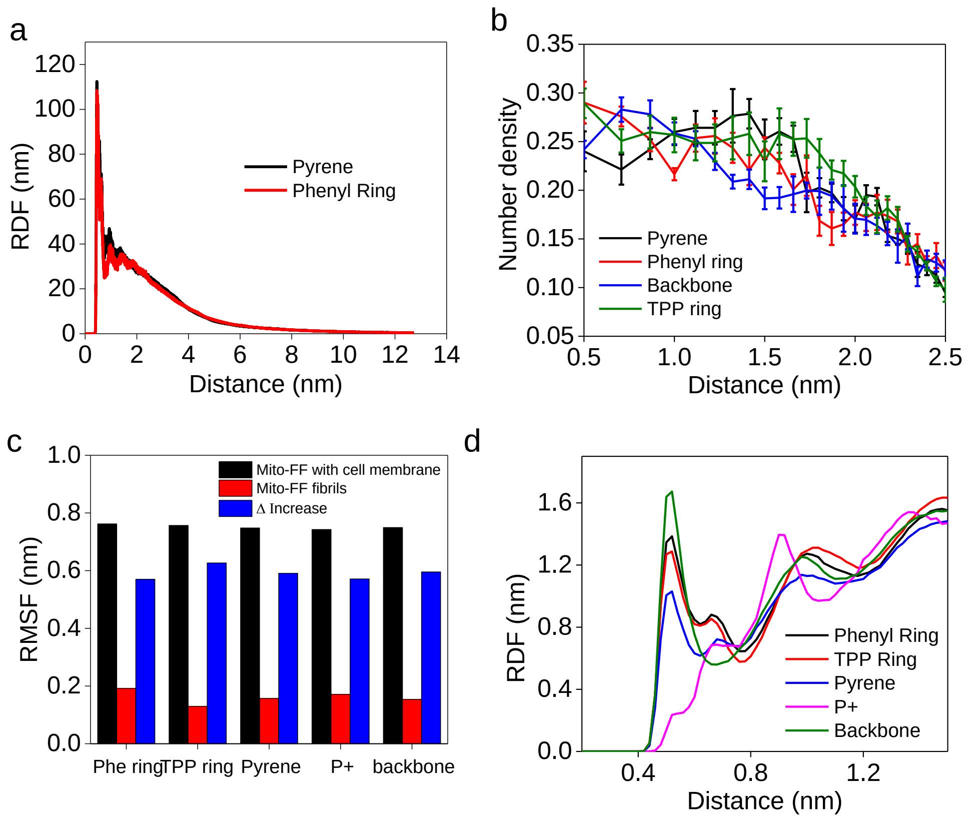

Supplementary Figure 22 | Coarse-grained model of **Mito-FF**. (a) Radial distribution functions of pyrene and phenyl ring of Mito-FF showing 0.5 nm phenyl-pheyl and pyrene-pyrene inter-distances. (b) Radial number density of constituent molecules of Mito-FF from the center of fiber to the surface. Density was calculated by the number of groups in the same volume of radial shell. (c) Root mean square fluctuation (RMSF) of constituent molecules of **Mito-FF** in the cylindrical assembly. (d) Radial distribution function (RDF) of constituent molecules of **Mito-FF** in the cylindrical assembly with the cell membrane.

In the assembly surface of **Mito-FFs**, we observed structured pyrene stacking and di-phenyl, where their self-interdistances were about ~0.5 nm estimated by the positions of the first peaks from radial distribution functions (i.e. g(r)). Time-resolved configurations of simulation revealed that di-phenyl played a major role to induce the fibrous structure due to their pi stacking and hydrophobic interactions, where the fibrous assembly consisted of well distributed hydrophobic and hydrophilic components. Indeed, the radial number densities of pyrene, phenyl ring, backbone, and TPP were similar. To identify the main reason of the pore generation by the assembly, we have traced molecular behavior of each components of **Mito-FF** via root mean square fluctuation (RMSF) and radial distribution function (RDF).

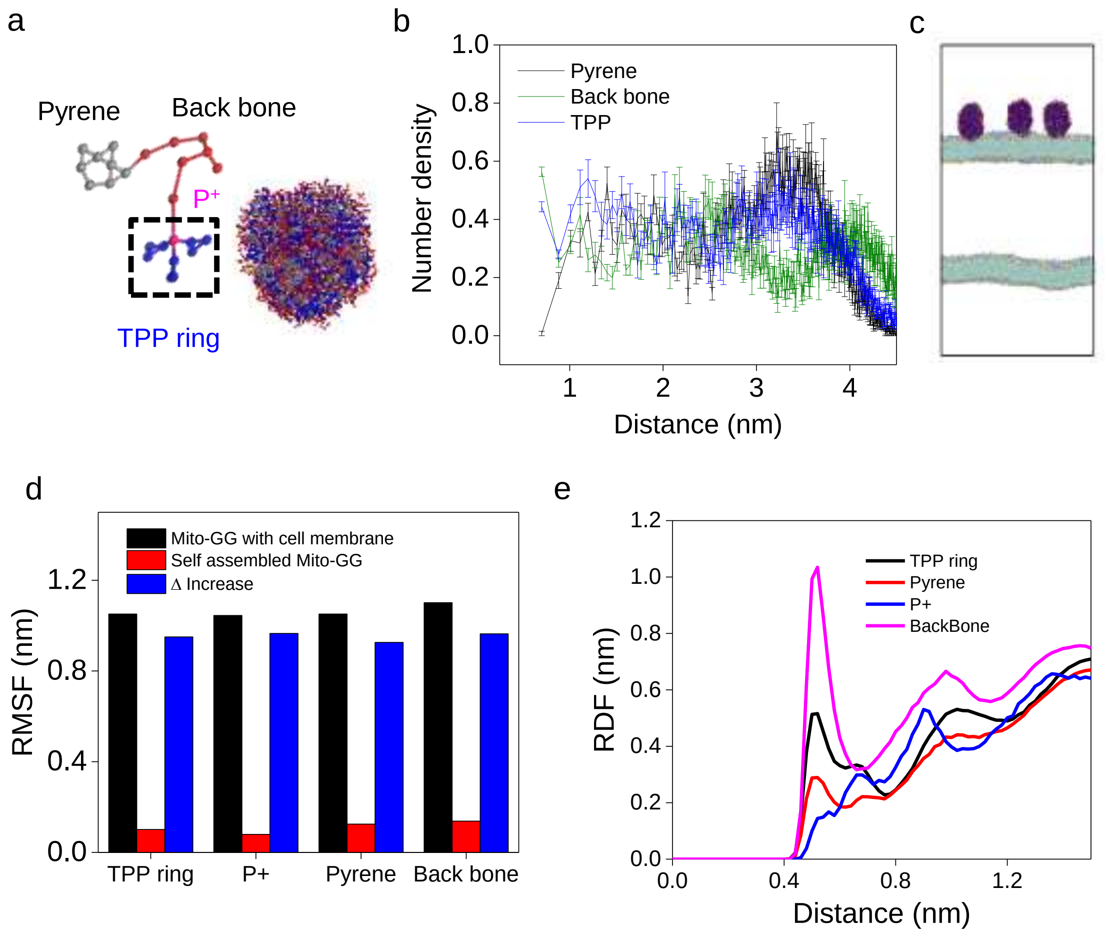

Supplementary Figure 23 | (a) Coarse-grained model of **Mito-GG** and self-assembled structure of **Mito-GGs**. TPP, back bone and pyrene groups consist of ten, seven and eight coarse-grained beads, respectively. Red, gray, blue and dark pink represent backbone, pyrene, benzene and P^+-C-C, respectively. (b) Radial number density of constituent molecules of **Mito-GG** from the center of sphere to the surface. Density was calculated by the number of groups in the same volume of spherical shell. (c) **Mito-GG**s on the cell membrane. Water and ions are omitted for a clear view. (d) Root mean square fluctuation (RMSF) of constituent molecules of **Mito-GG** in the spherical assembly. Notations in x-axis are indicated in (a). (e) Radial distribution function (RDF) of constituent molecules of **Mito-GG** in the spherical

assembly with the cell membrane.

Mito-GGs were self-assembled the spherical assembly via CGMD. Effective radius of the assembly was about ~4.5 nm. As described in the case of Mito-FF previously, **Mito-GGs** were assembled to be a spherical shape without di-phenyl groups but contained structured pyrene stacking. Like the cylindrical assembly of **Mito-FFs**, hydrophobic (pyrene groups) and hydrophilic (TPP groups) components comparably existed inside and in the vicinity of the surface of the assembly, respectively, as shown in **Figure 16 b.** Unlike the case of the cylindrical assembly, the density of backbone was higher than other groups in Mito-GG in the vicinity of the surface of the assembly. The existence of backbone at the surface of the sphere assembly is considered lowering the zeta potential of TPP. In order to investigate the penetrability of the spherical assembly, CGMD simulation was performed with three assembled **Mito-GGs**, which were located above cell membrane (**Figure 16c**). No holes were generated by the assemblies. As seen from **Mito-FFs**, RMSFs of each component of **Mito-GG** were increased. Backbone groups were fluctuated most highly due to high surface density. When compared with the self-assembly model, P^+ ions and backbone showed the large increase of RMSF. Moreover, RDF showed a similar trend of TPP rings and pyrene as in the case of **Mito-FFs** with the high peak of backbone. Other groups showed smaller intensity of peaks, which represent less coverage of benzene over the cell membrane. The results infer that the phenyl rings could help to overcome the energy barrier required for the penetration of cell membrane. The spherical assembly, however, did not have enough benzene groups to induce the penetration. Notably, the penetrability was further reduced by the blockage of backbone on the interaction of sphere assembly with cell membrane.

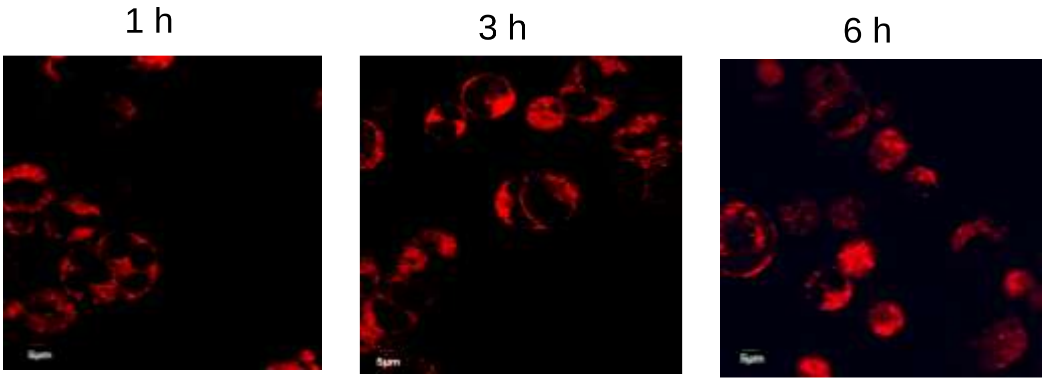

Supplementary Figure 24 | Mitochondrial membrane depolarization analysis of HEK 293 T cells measured with TMRM after treating with **Mito-FF** at different time point showed no mitochondrial membrane depolarization.

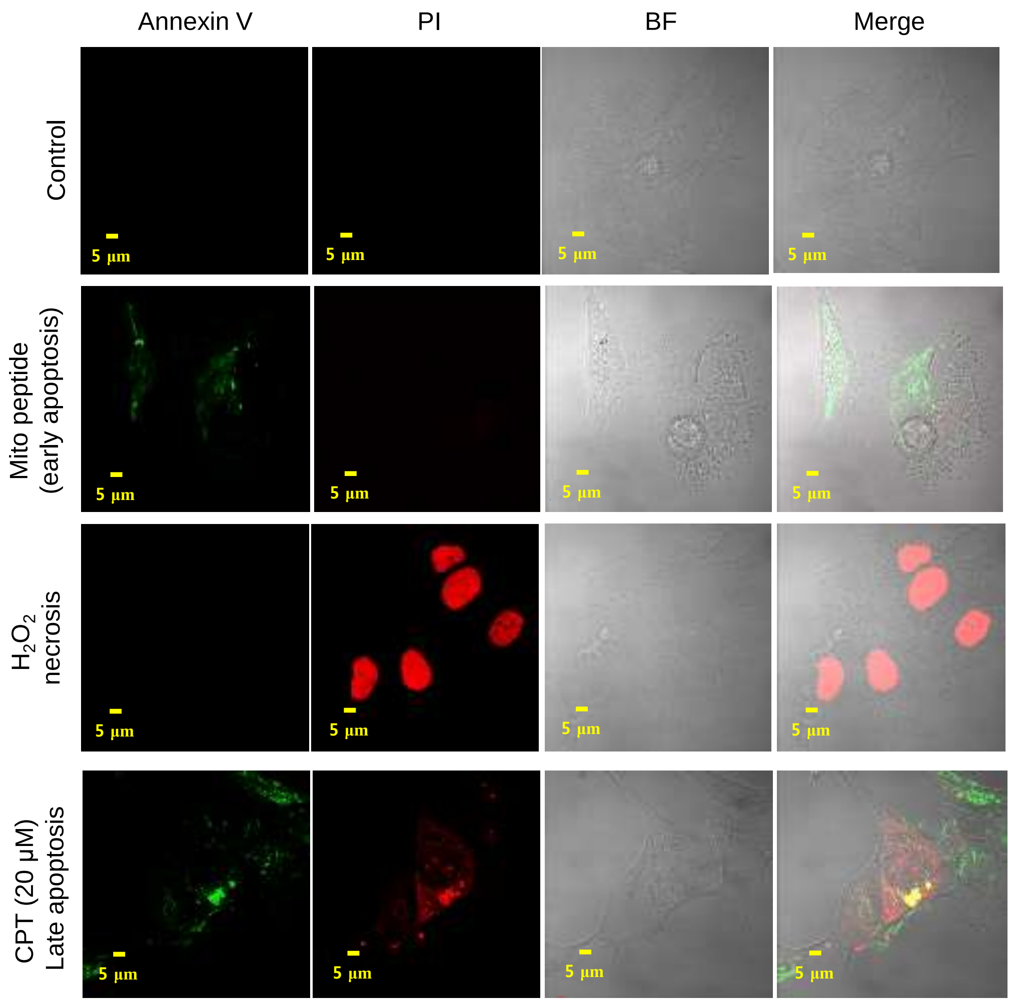

Supplementary Figure 25 | Confocal images with Annexin/PI staining on HeLa cells. Images show no staining by Annexin V and PI in the live cells (first raw). +Annexin V, -PI staining after treating with Mito-FF (second raw) .-Annexin, +PI after inducing necrosis by H2O2 (third raw) and +Annexin, +PI after inducing late apoptosis by Camptothecin (10 μM) (fourth raw) in the HeLa cells for an incubation period of 12 h. After treating with **Mito-VV** or **Mito-FxFx** also showed green fluorescence appearance, indicating that cells entered in early apoptosis within 6 h.

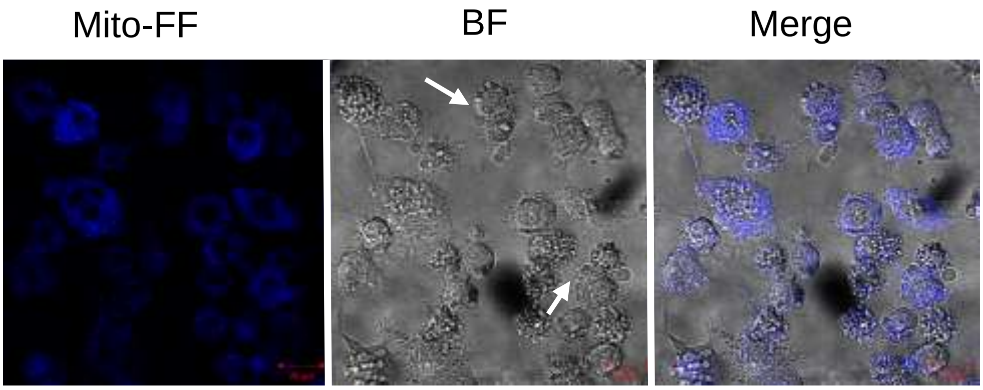

Supplementary Figure 26 | The confocal microscopy analysis showing the membrane blebbing of HeLa cells after treating with **Mito-FF**, suggesting that the cells underwent apoptosis.

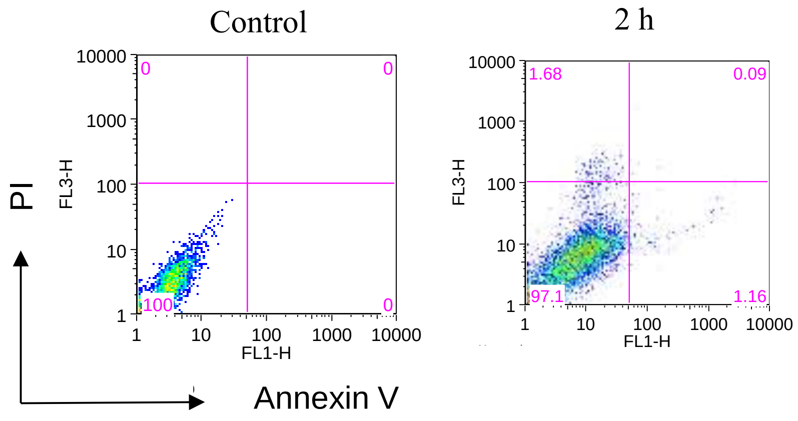

Supplementary Figure 27 | Flow cytometric analysis of **Mito-FF** were obtained with Annexin/PI staining on HeLa cells after treating with **Mito-FF** for desired time point. Control experiment was performed without **Mito-FF**.

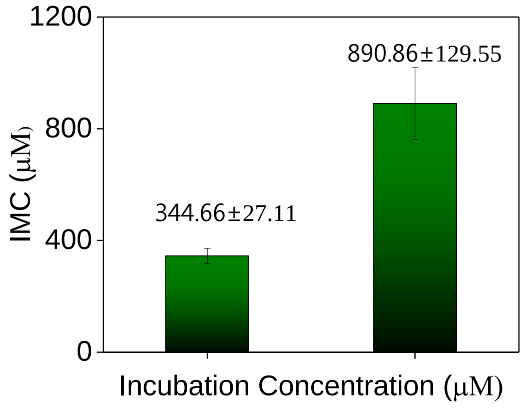

Supplementary Figure 28 | Mitochondrial accumulation of **Mito-FF** in HEK293T cells showed the about 4 fold decrease in the mitochondrial accumulation in HEK293T cells compared to HeLa cells. Data represent mean ± s.d from three independent experiments.

Supplementary Methods.

1.1 Spectrofluorometric analysis of Mito-FF.

Mito-FF solution (100 μM and 10 μM) was prepared in PBS (10 mM) and MeOH (HPLC grade). The fluorescence emission was measured for each solution separately with an excitation wavelength of 343 nm (absorption maxima detected form UV-Visible spectrometer) at room temperature, and emission were detected from 360 – 600 nm (slit width 2.5) using Fluorescence Spectrophotometer (F-7000, HITACHI).

1.2 FT-IR Analysis of Mito-FF.

Mito-FF solution (1 mM) was prepared in water. The samples were freeze-dried for one day. The collected **Mito-FF** powder was analyzed using FT-IR spectrometer (VARIAN) in ATR mode.

1.3 Transmission Electron Microscopic analysis of Mito-FF.

A drop of the aqueous solution of **Mito-FF** was placed on a formvar/carbon-coated copper grid and allowed to evaporate under ambient conditions. The sample was stained with 2 wt% uranyl acetate solution, allowed to evaporate for 1 min and excess solution removed with a filter paper. The specimen was observed with JEM-1400 TEM operating at 120 kV. Structural

information of self-assembled **Mito-FF** fibrils was obtained with over 100 nanofibers of more than 5 micrographs by simple measure program (JEOL Ltd., Tokyo, Japan).

1.4 Determination of the concentration of Mito-FF/Mito-FF-NBD inside the mitochondria of HeLa cells.

To calculate the concentration of Mito-peptides inside mitochondria, HeLa cells and HEK 293 T cells were seeded in a 24 well plate (Thermo Fisher Scientific Inc). The cells were allowed for attachment for 24 h at 37 ºC, 10% CO_2. The cultural medium was removed and new medium containing 5 µM and 10 µM of **Mito-FF/** 10 µM and 100 µM of **Mito-FF-NBD** and allowed to have sufficient cellular uptake by incubating at 37 ºC. After 3 h, the medium were removed, cells were washed with fresh medium (two times) followed by cold 1X PBS. Using trypsin solution, HeLa cells were harvested from the surface and collected in a falcon tube. The average cell number was determined using LUNAR cell counter. The cells were centrifuged with 1400 rpm for 5 min, collected as a pellet, and supernatant discarded. The pellet gently suspended in PBS and centrifuged again, and supernatant discarded. The cells were collected as a pellet and 200 µL of Ripa cell lysis buffer were added and waited for 30 min, after ensuring the complete lysis of the cell, 200 µL of MeOH were added for the clarity of the spectra. The supernatant was collected by centrifugation and used for the experiment. The fluorescence emission spectra for these samples were measured (for **Mito-FF** samples were excited at 343 nm wavelength and for **Mito-FF-NBD** samples were excited at 465 nm). A calibration curve was plotted initially for the **Mito-FF/Mito-FF-NBD** at different concentrations ranging from 0.03 µM to 1 µM in 1:1 mixture of buffer and MeOH (200 µL of MeOH and 200 µL of Ripa lysis buffer) and plotted a calibration curve with value of measured fluorescence intensity against concentration. Using this calibration plot, the concentration of **Mito-FF/Mito-FF-NBD** in each of the collected supernatant was determined, which provide the amount of sample uptaken by the cell. The intracellular concentration was calculated by using the relationship;

*Intracellular concentration = cellular uptake (µmol) / Cell number x Volume of the HeLa cell.

The average size of the HeLa cell is 20 µm, and the average cell volume for single HeLa cell

were calculated as 4000 µm^3.We have then determined the mitochondrial concentration using the relation of the volume ratio of HeLa cell to the HeLa cell mitochondria as 0.06.

1.5 Two photon microscopic analysis of Mito-FF in HeLa cell.

The HeLa cells were cultured in 8 well Lab Tek II slide chamber at 80% confluence in DMEM (Life Technologies) supplemented with 10% FBS and 1% penicillin/streptomycin at 37°C and 5% CO_2. The cells were incubated with **Mito-FF**, 20 µm for 1 h. The images were taken using multi photon LSM-780 confocal microscopy (UOBC center, UNIST). After finding the correct focus, the sample at 780 nm wavelength two photon laser, and emission were observed via band pass filter range form 420 – 480 nm. The filter was then changed with 380 – 420 nm with disturbing the initial set-up, and snap shots were taken. The raw data were collected and analyzed via METLAB.

1.6 Molecular Dynamics Methods.

In order to perform coarse-grained molecular dynamics (CGMD) simulation, a CG force field is required. Martini force field was developed by Marrink *et al* for lipid membrane, and recently, the coverage of the force field has been extended to proteins and carbon-based molecules[1,2]. The coarse-graining basic rule in Martini force field is that two to four carbon-compositions are coarse-grained to one bead. For example, C_4H_{10} can be represented as one bead and benzene ring can be represented as three beads, where each bead contains C_2H_2 group. To incorporate Martini force field to our system, **Mito-FF-NBD**, **Mito-FF** and **Mito-GG** were coarse-grained by following the rule of Martini as shown in Supplementary Figs. 9a, 9b and 16a, respectively. For the self-assembly simulations of **Mito-FFs** and **Mito-GGs**, 243 molecules were used in the box of 25×25×30 nm^3 and 30×30×30 nm^3, respectively, which were filled with water. The simulations were run for 5 μs.

For the cell membrane system with **Mito-FF** as shown in Fig. 5c, the bilayer was constructed with 3500 DPPCs and 1500 DPPGs (i.e. 2.3:1)[3]. We put two bilayers to separate inside and outside of the cell, where 5657 Cl^- ions were introduced between the bilayers to incorporate a low pH environment (i.e ~3)[4].The distance between two bilayers was set to 30 nm and three **Mito-FFs** were introduced at 1 nm above the membrane. For the cell membrane system with **Mito-GG** as shown in Supplementary Fig.16c, the bilayer was constructed with 5380 DPPCs and 2306 DPPGs (i.e. 2.3:1)[3]. In this system, 8450 Cl^- ions were used to incorporate a low pH

environment (i.e ~3)[4]. The distance between two bilayers was set to 35 nm and three **Mito-GGs** were introduced at 1 nm above the membrane. The box sizes of the cell membrane systems with **Mito-FFs** and **Mito-GGs** were 30×30×72 nm^3 and 50×50×80 nm^3, respectively. The simulations were run for 2 μs and 1.56 μs for **Mito-FF** and **Mito-GG** systems, respectively. Note that CG models for DPPC and DPPG in the membrane system were taken from Tian and Ma's work[5].

All CGMD simulations were performed at 300 K and 1 bar with Berendsen thermostat and barostat. For the cell membrane systems, semi-isotropic pressure coupling was used in the perpendicular direction to the cell membrane. The cut off radius of van der Waals and short-ranged Coulombic interaction were set to 1.2 nm. The time step was set to 20 fs. For running CGMD simulations, GROMACS 5.0.3 package was used[6].

1.7 TEM sample preparation for the visualization of Mito-FF fibrils inside isolated mitochondria.

The mitochondria were isolated by reported protocol[7]. 100 μg of the mitochondria were incubated with 10 μM of **Mito-FF** at 37 ^{o}C in mitochondria incubation buffer, after 30 min mitochondria were centrifuged and further processed for TEM imaging. The similar procedure was conducted without **Mito-FF** for the control mitochondria TEM imaging.

1.8 TEM sample preparation protocol.

HeLa cells were grown in 24 well plate on 15 mm diameter Theramanox® coverslips (Nalge Nunc International, NY) in 1 mL serum contain media with 80 % confluency. The cells were treated with 20 μM of **Mito-FF** or **Mito-GG** for 3 h. The cells were then fixed using 2% glutaraldehyde for 30 min at room temperature and then washed with 0.1M Na-PO4 buffer + 5% sucrose (1 mL), 3x over 30 min at room temperature. The TEM samples of the cell were prepared according to the reported protocol.

1.9 TEM analysis of Mito-FF fibrils within the isolated mitochondria.

The specimen of Mito-FF fibrils within the isolated mitochondria was observed with a JEM-1400 operating at 120 kV. Structural information of self-assembled Mito-FF was provided with more than 100 nanofibers of over 10 micrographs by Simple Measure program (JEOL Ltd., Tokyo, Japan).

1.10 TEM Tomography (TEMT) of mitochondria with Mito-FF fibrils.

3D reconstruction of mitochondria obtained from a series of 2D image projections of the samples at different viewing angles. For TEMT, images were obtained using a JEM-1400 (JEOL Ltd., Tokyo, Japan), operating at 120 kV, and a charge-coupled device (CCD) camera size of 1046 × 1046 (JEOL Ltd., Tokyo, Japan). In total, 137 images were acquired at tilting angles between -68° and +68°, with an increment of 1°. The magnification was x 30K, corresponding to a pixel size of 0.85 nm. Tilting, refocusing, and repositioning were carried out after every individual tilt increase. Alignment and reconstruction of tilt series were performed in IMOD software. UCSF chimera software was used for visualization.

1.11 Model membrane preparation and dye leakage assay

Large unilamellar vesicles (LUV) of L-α-Phosphatydyl-DL-glycerol (PG) (Sigma aldrich)/Cholesterol (Alfa Aeser) 7/3, were prepared from a chloroform solution of lipids in the desired ratio. The solution was gently dried under evaporator and then placed under a high vacuum overnight to further evaporate any residual solvent. The obtained lipid film was rehydrated with a buffer solution (10 mM phosphate buffer solution, pH 7.4, 100 mM NaCl), to yield a final concentration of 15 mM and dispersed by vigorous stirring[7]. To prepare dye-filled LUV, the dry lipid film was hydrated with a buffer solution containing calcein (10 mM phosphate buffer solution, 80mM calcein, pH 7.4) to a final concentration of 10 mg/ml. To remove non encapsulated calcein, the LUV containing solution were passed through Sephadex 50 gel exclusion column (Sigma-Aldrich) and collected the first colored detectable band under visible light. Solutions were freshly prepared before experiments and use directly. For the dye leakage experiments, the calcein filled LUV composed of PG/Cholesterol bilayer membrane. Membrane leakage was detected by measuring the increase in calcein fluorescence resulting from membrane leakage. The dye-filled vesicles solution were diluted about 10 times with buffer solution (10 mM phosphate buffer solution, pH 7.4) and used for the experiments. To initiate membrane disruption, **Mito-FF** peptide stock solution was added to 200 μL of the LUV solution to obtain a final peptide concentration of 10 or 500 μM respectively. The fluorescence increment was recorded using a Fluorescence

Spectrophotometer (F-7000, HITACHI). The procedure was repeated for **Mito-GG**.

1.12 Protein Analysis

The matrix-APEX2 plasmid was introduced into HeLa cells by Lipofectamine 2000 (Invitrogen, 11668-019). After 24 h (transfection), the medium was changed to fresh medium containing **Mito-FF** or **Mito-GG** for 4 h in the incubator. Then, cells were washed with two times of fresh medium and changed again to 500 µM biotin-phenol containing fresh medium for 30 min. After that, the medium was replaced by 1 mM H_2O_2 in DPBS and washed by DPBS three times. Then, cells were fixed by 4% of *p*-formaldehyde for 15 min and washed by DPBS two times. Permeabilization of cells was performed by methanol for 5 min in -20 °C and washed again by DPBS two times. After permeabilization, cells were treated by blocking solution (2% dialyzed BSA) for 30 min at room temperature. To detect APEX2 protein expression, cells were incubated with mouse anti-V5 antibody (Invitrogen, cat. no. R960-25, 1:3000 dilution) in blocking solution for 30~60 min at room temperature. Then, cells were washed with TBST each 5 min. After washing, cells were simultaneously incubated with anti-mouse-Alexa Fluor 488 (Invitrogen, cat. no. A-11001, 1:1000 dilution) in blocking solution for 30 min room temperature. After cells were washed with TBST each 5 min again, cells were imaged by were taken using a Carl Zeiss LSM780NLO confocal laser scanning microscope (Jena, Germany) in UNIST Olympus Biomed Imaging Center, Ulsan.

1.13 Zeta potential analysis of Mito-FF and Mito-GG.

200 µM (above the CAC) of **Mito-FF** and Mito-GG solutions in water was prepared and analyzed the surface charge using Malvern Zetasizer ZS series (United Kingdom). Three measurements were done and the average of three measurements were taken as the surface charge.

Reference.

1 Marrink, S. J., Risselada, H. J., Yefimov, S., Tieleman, D. P. & Vries, A. H. The MARTINI force field: coarse grained model for biomedical simulations. *J. Phys. Chem.* B **111**, 7812 – 7824 (2007).

2 Jong, D. H., Singh, G., Bennett, W. F. D., Arnarez, C., Wassenaar, T. A., Schafer, L. V., Periole, X., Tielman, D. P & Marrink, S. J. Improved parameters for the martini

coarse-grained protein force filed. *J. Chem. Theory Comput* **9**, 687 – 697 (2013).

3 Lin, J., Zhang, H., Chen, Z. & Zheng, Y. Penetration of lipid membranes by gold nanoparticles : Insights into cellular uptake, cytotoxicity, and their relationship. *ACS Nano* **9**, 5421-5429 (2010).

4 Liu, F. F., Huang, B., Yan, X. & Sun, Y. Molecular basics for the dissociation dynamics of protein A-immunoglobulin G 1 complex. *PLOS one* **8**, e66935 (2013).

5 Kang, B. H., Plescia, J., Dohi, T., Rosa, J., Doxsey, S. J. & Altieri, D. C. Regulation of tumor cell mitochondrial homeostasis by an organelle-sopecific Hsp90 chaperone network. *Cell*, **131**, 257-270 (2007).

6 Kim, C., Agasti, S. S., Zhu, Z., Isaacs, L. & Rotello, V. M. Recognition-mediated activation of therapeutics gold nanoparticles inside living cells. *Nat. Chem.* **2**, 962-966 (2010).

7 Sciacca, M. F., Kotler, S. A., Brender, J. R., Chen, J., Lee, D. K. & Ramaswamy, A. two-step mechanism of membrane disruption by Aβ through membrane disruption fragmentation and pore formation. *Biophys. J.* **103**, 702-710 (2012).
